# Development of a Prediction Model for the Management of Noncommunicable Diseases Among Older Syrian Refugees Amidst the COVID-19 Pandemic in Lebanon

**DOI:** 10.1001/jamanetworkopen.2022.31633

**Published:** 2022-10-13

**Authors:** Stephen J. McCall, Tanya El Khoury, Noura Salibi, Berthe Abi Zeid, Maria El Haddad, Marwan F. Alawieh, Sawsan Abdulrahim, Monique Chaaya, Hala Ghattas, Abla M. Sibai

**Affiliations:** 1 Center for Research on Population and Health, Faculty of Health Sciences, American University of Beirut, Beirut, Lebanon; 2 Norwegian Refugee Council (NRC), Beirut, Lebanon; 3 Department of Health Promotion and Community Health, Faculty of Health Sciences, American University of Beirut, Beirut, Lebanon; 4 Department of Epidemiology and Population Health, Faculty of Health Sciences, American University of Beirut, Beirut, Lebanon

## Abstract

**Importance:**

Older Syrian refugees have a high burden of noncommunicable diseases (NCDs) and economic vulnerability.

**Objectives:**

To develop and internally validate a predictive model to estimate inability to manage NCDs in older Syrian refugees, and to describe barriers to NCD medication adherence.

**Design, Setting, and Participants:**

This nested prognostic cross-sectional study was conducted through telephone surveys between September 2020 and January 2021. All households in Lebanon with Syrian refugees aged 50 years or older and who received humanitarian assistance from a nongovernmental organization were invited to participate. Refugees who self-reported having chronic respiratory disease (CRD), diabetes, history of cardiovascular disease (CVD), or hypertension were included in the analysis. Data were analyzed from November 2021 to March 2022.

**Main Outcomes and Measures:**

The main outcome was self-reported inability to manage any NCD (including CRD, CVD, diabetes, or hypertension). Predictors of inability to manage any NCD were assessed using logistic regression models. The model was internally validated using bootstrapping techniques, which gave an estimate of optimism. The optimism-adjusted discrimination is presented using the *C* statistic, and calibration of the model is presented using calibration slope (*C* slope).

**Results:**

Of 3322 older Syrian refugees, 1893 individuals (median [IQR] age, 59 [54-65] years; 1089 [57.5%] women) reported having at least 1 NCD, among whom 351 (10.6% overall; 18.6% of those with ≥1 NCD) had CRD, 781 (23.7% overall; 41.4% of those with ≥1 NCD) had diabetes, 794 (24.1% overall; 42.2% of those with ≥1 NCD) had history of CVD, and 1388 (42.3% overall; 73.6% of those with ≥1 NCD) had hypertension. Among individuals with NCDs, 387 participants (20.4%) were unable to manage at least 1 of their NCDs. Predictors for inability to manage NCDs were age, nonreceipt of cash assistance, household water insecurity, household food insecurity, and having multiple chronic diseases, with an adjusted *C* statistic of 0.650 (95% CI, 0.620-0.676) and *C* slope of 0.871 (95% CI, 0.729-1.023). The prevalence of nonadherence to medication was 9.2%, and the main reasons for nonadherence were unaffordability of medication (40.8%; 95% CI, 33.4%-48.5%) and the belief that they no longer required the medication after feeling better (22.4%; 95% CI, 16.4%-29.3%).

**Conclusions and Relevance:**

In this cross-sectional study, the predictors of inability to manage NCDs among older Syrian refugees in Lebanon were mainly related to financial barriers. Context-appropriate assistance is required to overcome financial barriers and enable equitable access to medication and health care.

## Key Points

**Question:**

What are the predictors and barriers to managing noncommunicable diseases (NCDs) for older Syrian refugees in Lebanon?

**Findings:**

This prognostic study including 1893 refugees with at least 1 NCD (chronic respiratory disease, diabetes, history of cardiovascular disease or hypertension) developed a predictive model for the inability to manage any NCD with a moderate discriminative ability. Predictors of inability to manage any NCD included age, no cash assistance, household water and food insecurity, and having multiple chronic diseases.

**Meaning:**

These findings suggest context-appropriate assistance is required to overcome financial barriers and enable equitable access to health care and medication required to manage NCDs among refugees.

## Teaser

This cross-sectional study develops and validates a model to estimate risk of inability to manage noncommunicable diseases in older Syrian refugees in Lebanon.

## Introduction

Forcibly displaced populations have been recognized as vulnerable to the direct and indirect impacts of the COVID-19 pandemic.^1,2,3^ Furthermore, older refugees are particularly vulnerable since they have a higher prevalence of preexisting chronic diseases^4^ and have an increased risk of hospitalization and mortality due to COVID-19.^5^ Before the COVID-19 pandemic, poor management of noncommunicable diseases (NCDs) was a global public health concern and is a neglected area in humanitarian emergencies.^6,7,8^ NCD self-management, including lifestyle changes and adherence to medication, has been shown to be associated with reduced premature mortality and morbidity.^9,10,11^ However, in refugee populations, NCD management is challenging owing to the competing demands of basic needs, such as access to adequate food, shelter, protection, and water. A study conducted in 2018 on Palestinian refugees in Lebanon found that 30% of the sample had low adherence to medications.^12^ Furthermore, refugee populations in Lebanon may have difficulty in adherence owing to poor access to health services, unavailability of medications, unaffordability of transportation, being food insecure, having limited health literacy, and other stressors.^13,14^

In Lebanon, large populations of Syrian refugees are situated in the Bekaa valley and North Lebanon and reside in informal tented settlements or residential areas.^15^ Before 2011, the Lebanese health system within these areas was already stretched to meet the needs of the host population.^13,14^ Syrian refugees have free access to chronic disease medication through the United Nations High Commissioner for Refugees (UNHCR) or other nongovernmental organizations.^16^ However, the COVID-19 pandemic and the recent major economic crisis in Lebanon have impacted access to health care and supply of essential medications,^17,18^ which may have a negative impact on the health status of Syrian refugees.^19,20^

Studies on NCD management among refugees in Lebanon and the Middle East, a region that hosts one of the largest refugee populations, are scarce.^12^ Understanding the barriers and predictors of self-reported management of NCDs among older refugees, a population at high risk for severe COVID-19 outcomes, is important to allow resource allocation and contextualized humanitarian assistance to prevent premature mortality and morbidity. The present study aimed to elucidate the predictors of the inability to manage NCDs in older Syrian refugees and describe barriers to accessing health care and managing these chronic conditions.

## Methods

This prognostic study was reviewed and approved by the American University of Beirut Social and Behavioral Sciences Institutional Review Board. Verbal consent was obtained from all study participants. This study is reported following the Transparent Reporting of a Multivariable Prediction Model for Individual Prognosis or Diagnosis (TRIPOD) reporting guideline and Strengthening the Reporting of Observational Studies in Epidemiology (STROBE) reporting guideline.

### Study Design, Sample, and Study Population

This was a cross-sectional study nested within a multiwave longitudinal study that aimed to examine the vulnerabilities of older Syrian refugees residing in Lebanon during the COVID-19 pandemic.^21^ The study included Syrian refugees aged 50 years and older who were identified from a full listing of beneficiary households of a nongovernmental humanitarian organization (the Norwegian Refugee Council).

Within the beneficiary sampling frame, all households who had used services offered by the humanitarian organization between 2017 and 2020 and included an adult aged 50 years or older were contacted and were included in the study. If there were multiple adults aged 50 years or older within a household, 1 person was randomly selected, invited to participate, and provided oral consent to enter the study. Individuals aged 65 years or older were assessed for capacity to participate using 5 modified items from the University of California, San Diego, Brief Assessment of Capacity to Consent (eMethods in the Supplement).^22^ The same respondent was approached to complete a telephone survey across different time points. For this study, the demographic characteristics were extracted from waves 1 (September-December 2020) and data related to NCDs and health were extracted from waves 2 (October 2020-January 2021).

### Data Sources

The questionnaire for each wave was developed using a combination of sources, including validated questionnaire modules, contextually specific questions, and community-identified priorities. The survey was cocreated by academics, humanitarian actors, local government officials, and focal points from the refugee communities. The questionnaire was piloted internally with data collectors and local community focal points to ensure face validity.^23^ Trained data collectors administered the surveys in Arabic and entered data into structured electronic data collection forms hosted on KoBoToolbox. Data entry checks and monitoring were performed for quality assurance. More details are available in eMethods in the Supplement.

### Outcome Measures

The outcome variable was self-reported inability to manage any NCD. This included the following conditions: cardiovascular disease (CVD), chronic respiratory disease (CRD), diabetes, or hypertension. For each condition, participants were asked “Are you able to manage your [condition]?”

### Candidate Predictors

Using the literature, 16 potential predictors were identified for inability to manage NCDs and were included for model development. These included age, sex, residence, education, smoking status, number of chronic conditions, hypertension, diabetes, CRD, CVD, living arrangement, food insecurity (measured using the Food Insecurity Experience Scale^24^), employment status, water insecurity (measured using the short-form Household Water Insecurity Scale^25^), receipt of cash assistance, and family debts.

### Missing Data

The largest amount of missing data in any variable was 1.8%. Missing data were assumed to be missing at random, so we used complete case analysis.^26^

### Statistical Analysis

Absolute frequencies and proportions are presented alongside odds ratios (ORs) with their 95% CIs using unadjusted logistic regression models, which examined the odds of inability to manage any NCD for each candidate predictor. This study used 2-sided tests with a statistical significance level of 5%.

All variables were categorical except age, which was found to have a linear association with inability to manage NCD. All candidate predictors of inability to manage NCD were entered into multivariable logistic regression models and removed using a stepwise backward method using a *P* < .157.^27^ This has been used as a proxy for the Akaike Information Criterion (AIC), in which predictors are removed to obtain the lowest AIC. Multicollinearity was assessed using correlation matrices and variance inflation factor (VIF); a VIF greater than 5 indicated collinearity. Number of NCDs and each chronic condition were modeled in 2 separate models. Smoking status was removed from the final model to improve model fit and prevent overfitting.^28^

The final model’s performance in terms of discrimination was assessed using the *C* statistic, which ranges from 0.5 to 1.0 (this is also known as the area under the receiver operating characteristic curve [ROC]), in which a value of 1.0 represents perfect discriminative ability between those with and without the outcome and 0.5 denotes a discriminative ability equal to chance. We also assessed the calibration of the final model, which describes the agreement between observed outcomes and predictive probabilities.^29^ This was assessed using calibration plots, which categorizes patients into 10 groups according to predictive probabilities, where the mean predicted risk within each of these groups is plotted against the mean observed proportion of events.^29,30^ If there is a perfect calibration, the graph will show a diagonal line where there is a slope of 1 and intercept of 0. A slope less than 1 suggests overfitting in the model, meaning that respondents with high risk of the outcome have overestimated risk predictions while those with low risk of the outcome have underestimated risk predictions. Moreover, we assessed whether there was an overall difference between the observed number of events and the average predictive risk using the calibration-in-the-large (CITL).

The final model’s selection of predictors, discrimination, and calibration estimates were internally validated using bootstrap methods, in which 500 bootstrap samples with replacement were used to validate the model selection process and generate an estimate of optimism, an optimism-adjusted estimates of *C* statistic, and an optimism-adjusted calibration plot.^26,29,31,32^ Bootstrap shrinkage was applied to the final apparent model, and the modified β coefficients and ORs are presented. A sensitivity analysis included modeling the total number of chronic diseases as a categorical variable. All analyses were conducted using Stata/SE statistical software version 17 (StataCorp). Data were analyzed from November 2021 to March 2022.

## Results

### Characteristics of the Population

**Figure 1.**
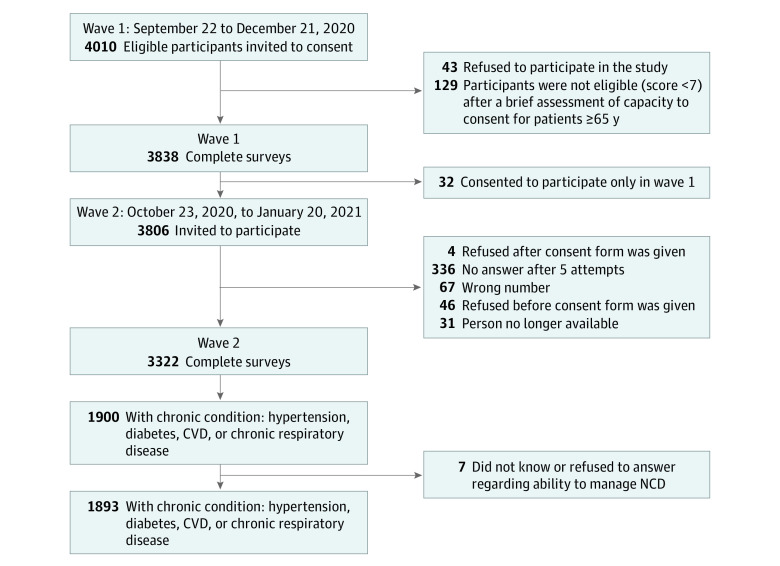
Flow Diagram of Syrian Refugees Included in the Study Population CVD indicates cardiovascular disease; NCD, noncommunicable diseases.

Of 17 384 households initially contacted at wave 1, 4010 eligible beneficiary Syrian refugees aged 50 years or older were invited to participate; of those, 3322 beneficiaries consented orally and participated in both waves 1 and 2 (Figure 1). Participants who were lost to follow-up had similar characteristics to wave 2; the study population had a larger proportion of women and had a higher median age than the all participants in wave 2 (eTable 1 in the Supplement).

Of 3322 older Syrian refugees who participated in the wider study, 1893 participants (median [IQR] age, 59 [54-65] years; 1089 [57.5%] women) reported having at least 1 NCD, 387 (20.4%) noted that they were unable to manage their condition through any means (lifestyle or medication) (Table 1). Among these participants, 174 were unable to manage their NCD through medication. Hypertension topped the listed of reported NCDs (1388 participants [42.3% overall; 73.6% of those with ≥1 NCD]), followed by CVD (794 participants [24.1% overall; 42.2% of those with ≥1 NCD]), diabetes (781 participants [23.7% overall; 41.4% of those with ≥1 NCD]), and CRD (351 participants [10.6% overall; 18.6% of those with ≥1 NCD]). The prevalence of NCDs among the entire sample is provided in eTable 2 in the Supplement; the ability to manage according to NCD type and number of NCDs is available in eTable 3 in the Supplement.

**Table 1.** Characteristics and Vulnerability Markers of Older Syrian Refugees Stratified by the Ability to Manage Their Noncommunicable Disease

Characteristic	Participants, No. (%)			Odds ratio (95% CI)^a^	*P* value
	Total (n = 1893)	Able to manage NCD (n = 1506)	Unable to manage NCD (n = 387)		
Age, median (IQR), y	59 (54-65)	59 (54-65)	58 (53-64)	0.98 (0.97 to 1.01)	.08
Sex					
Men	804 (42.5)	658 (81.8)	146 (18.2)	1 [Reference]	.03
Women	1089 (57.5)	848 (77.9)	241 (22.1)	1.28 (1.02 to 1.61)	
Head of household					
No	437 (23.1)	345 (79.0)	92 (21.0)	1 [Reference]	.72
Yes	1456 (76.9)	1161 (79.7)	295 (20.3)	0.95 (0.73 to 1.24)	
Governorate					
Beirut/Mount Lebanon	28 (1.5)	21 (75.0)	7 (25.0)	1.16 (0.48 to 2.78)	.73
Beqaa/Baalbek-Hermel	826 (43.6)	684 (82.8)	142 (17.2)	0.72 (0.56 to 0.93)	.01
North/Akkar	727 (38.4)	565 (77.7)	162 (22.3)	1 [Reference]	NA
South/Nabatieh	312 (16.5)	236 (75.6)	76 (24.4)	1.12 (0.82 to 1.53)	.46
Residence					
Inside informal tented settlements	689 (36.4)	552 (80.1)	137 (19.9)	1 [Reference]	.64
Outside informal tented settlements	1204 (63.6)	954 (79.2)	250 (20.8)	1.06 (0.84 to 1.33)	
Living arrangement					
Alone	34 (1.8)	27 (79.4)	7 (20.6)	1.01 (0.44 to 2.33)	.98
With others	1859 (98.2)	1479 (79.6)	380 (20.4)	1 [Reference]	
Level of education					
Elementary	443 (23.4)	354 (79.9)	89 (20.1)	0.93 (0.70 to 1.22)	.59
Never attended school	1013 (53.6)	797 (78.7)	216 (21.3)	1 [Reference]	NA
≥Preparatory and secondary	434 (23.0)	352 (81.1)	82 (18.9)	0.86 (0.65 to 1.14)	.29
Missing	3	3	0	NA	NA
Smoking status					
Current smoker	538 (28.4)	430 (79.9)	108 (20.1)	1 [Reference]	NA
Exsmoker	255 (13.5)	213 (83.5)	42 (16.5)	0.79 (0.53 to 1.16)	.22
Nonsmoker	1099 (58.1)	862 (78.4)	237 (21.6)	1.09 (0.85 to 1.41)	.48
Missing	1	1	0	NA	NA
Chronic conditions, No.					
1	481 (26.0)	415 (86.3)	66 (13.7)	1 [Reference]	NA
2	728 (39.3)	596 (81.9)	132 (18.1)	1.39 (1.01 to 1.92)	.04
≥3	642 (34.7)	466 (72.6)	176 (27.4)	2.37 (1.74 to 3.24)	<.001
Missing	42	29	13	NA	NA
Hypertension					
No	498 (26.4)	402 (80.7)	96 (19.3)	1 [Reference]	.44
Yes	1388 (73.6)	1098 (79.1)	290 (20.9)	1.11 (0.85 to 1.43)	
Missing	7	6	1	NA	NA
Cardiovascular disease					
No	1089 (57.8)	891 (81.8)	198 (18.2)	1 [Reference]	.005
Yes	794 (42.2)	608 (76.6)	186 (23.4)	1.38 (1.10 to 1.72)	
Missing	10	7	3	NA	NA
Diabetes					
No	1105 (58.6)	901 (81.5)	204 (18.5)	1 [Reference]	.02
Yes	781 (41.4)	602 (77.1)	179 (22.9)	1.31 (1.05 to 1.64)	
Missing	7	3	4	NA	NA
Chronic respiratory diseases					
No	1537 (81.4)	1259 (81.9)	278 (18.1)	1 [Reference]	<.001
Yes	351 (18.6)	244 (69.5)	107 (30.5)	1.98 (1.53 to 2.58)	
Missing	5	3	2	NA	NA
Family debts					
No	71 (3.8)	59 (83.1)	12 (16.9)	1 [Reference]	.46
Yes	1817 (96.2)	1445 (79.5)	372 (20.5)	1.27 (0.67 to 2.38)	
Missing	5	2	3	NA	NA
Receipt of cash assistance					
No	576 (30.5)	432 (75.0)	144 (25.0)	1.48 (1.17 to 1.87)	.001
Yes	1315 (69.5)	1073 (81.6)	242 (18.4)	1 [Reference]	
Missing	2	1	1	NA	NA
FIES Categorical household food insecurity					
Food security	148 (8.0)	133 (89.9)	15 (10.1)	1 [Reference]	.08
Mild to moderate food insecurity	1071 (57.6)	904 (84.4)	167 (15.6)	1.64 (0.94 to 2.86)	
Severe food insecurity	640 (34.4)	450 (70.3)	190 (29.7)	3.74 (2.14 to 6.55)	<.001
Missing	34	19	15	NA	NA
Household water insecurity experiences scale					
Household water insecurity	515 (27.3)	377 (73.2)	138 (26.8)	1.66 (1.31 to 2.11)	<.001
No household water insecurity	1372 (72.7)	1124 (81.9)	248 (18.1)	1 [Reference]	
Missing	6	5	1	NA	NA

Abbreviations: FIES, Food Insecurity Experience Scale; NA, not applicable; NCD, noncommunicable disease.

^a^ Dependent (outcome) variable is unable to manage chronic disease compared with able to manage chronic disease.

**Figure 2.**
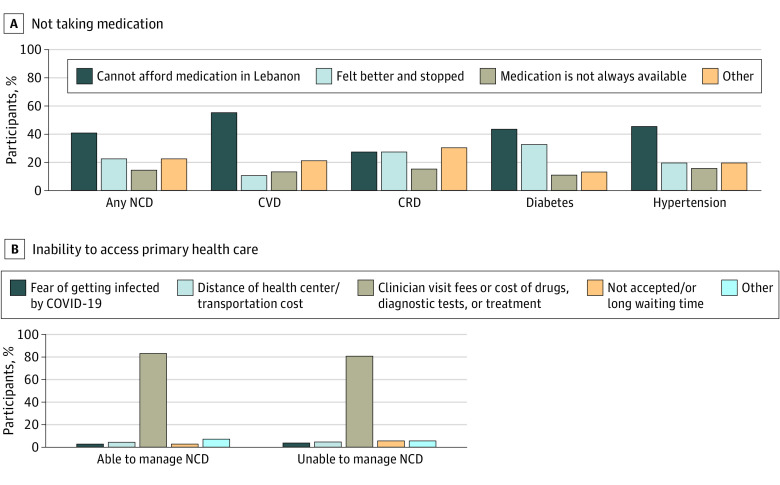
Primary Reasons for Not Using Medications or Accessing Primary Health Care Among Older Syrian Refugees A, Other reported reasons for not using medication included “I felt uncomfortable taking it and did not follow up with the physician,” “I used to get the medication from Syria, but owing to the closure of border crossings I am no more able to do so,” “I had allergy due to the medication,” and “I do not always feel sick.” B, Includes refugees who both were unable to access primary care and reported a noncommunicable disease (NCD; 293 participants). Other reported reasons for inability to access primary care included “do not know where to go,” “lack of documentation, center closed, or physician not available owing to COVID-19,” and “difficulty in transportation.” CRD indicates chronic respiratory disease; CVD, cardiovascular disease.

Among 174 participants who were unable to manage their NCD through medication, the primary reasons included feeling better and not needing the medication (22.4%; 95% CI, 16.4%-29.3%), medication not always available (14.4%; 95% CI, 9.5%-20.5%), and unaffordability of the medication (40.8%; 95% CI, 33.4%-48.5%). Unaffordability of medication remained the primary reason for CVD, diabetes, and hypertension when examining disease-specific reasons for nonadherence to medication (Figure 2A). In addition, there were 293 participants who were unable to access primary health care, among whom 109 participants were also unable to manage their NCD. Among these participants, the primary reason for being unable to access primary care was the cost of the doctor’s visits, medication or tests (80.7%; 95% CI, 72.1%-87.7%) (Figure 2B).

### Unadjusted Analysis

Unadjusted odds ratios of inability to manage NCD and 95% CIs of each potential predictor are reported in Table 1. Among older refugees, women were more likely than men to be unable to manage at least 1 NCD (OR, 1.28; 95% CI, 1.02-1.61), as were individuals with multiple chronic conditions (OR vs 1 chronic condition, 1.39; 95% CI, 1.01-1.92), those with a history of CVD (OR vs no CVD, 1.38; 95% CI, 1.10-1.72), those with diabetes (OR vs no diabetes, 1.31; 95% CI, 1.05-1.64), those with CRD (OR vs no CRD, 1.98; 95% CI, 1.53-2.58), those who did not receive cash assistance (OR vs no cash assistance, 1.48; 95% CI, 1.17-1.87), those with severe food insecurity (OR vs food secure, 3.74; 95% CI, 2.14-6.55), and those with household water insecurity (OR vs water secure, 1.66; 95% CI, 1.31-2.11).

### Predictors and Model Performance

**Figure 3.**
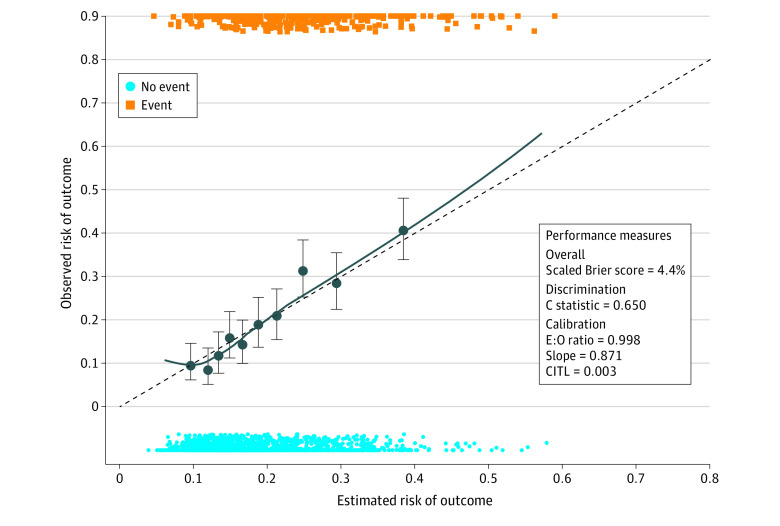
Model Performance in the Optimized-Adjusted Model The dots indicate mean observed and expected probabilities across the risk spectrum; whiskers, 95% CIs; and CITL, calibration-in-the-large.

The final model retained 8 predictors of inability to manage NCD, which included age; self-reported hypertension, diabetes, CVD, and CRD (the more NCDs an individual possessed, the higher the predicted risk of inability to manage them); severe household food insecurity; no receipt of cash assistance; and household water insecurity (Table 2). The final model had a moderate discriminative ability with an optimized adjusted *C* statistic of 0.650 (95% CI, 0.620 to 0.676) (Figure 3; eTable 4 in the Supplement). The calibration plot without correction for optimism is shown in eFigure in the Supplement, and the optimism-adjusted calibration plot is shown in Figure 3, which depicts the expected performance of the final model in future samples.^29,33^ The calibration slope after adjustment for optimism was 0.871 (95% CI, 0.729 to 1.023), the brier score was 4.4, and CITL was 0.003 (95% CI, –0.112 to 0.126) (Figure 3; eTable 4 in the Supplement). ORs and coefficients of the final model have been adjusted for overfitting and are presented in Table 2.

**Table 2.** Multivariable Model for Predicting Inability to Manage Any Noncommunicable Disease

Factor	Apparent model			Model adjusted by bootstrap shrinkage		
	Parameter estimate (95% CI)	OR (95% CI)	*P* value	Parameter estimate (95% CI)	OR (95% CI)	*P* value
Age	–0.01 (–0.03 to 0.00)	0.99 (0.97 to 1.00)	.14	–0.01 (–0.02 to 0.00)	0.99 (0.98 to 1.00)	.14
Self-reported medical history						
Cardiovascular disease^a^	0.22 (–0.02 to 0.46)	1.24 (0.98 to 1.58)	.08	0.19 (–0.02 to 0.40)	1.21 (0.98 to 1.49)	.07
Chronic respiratory disease^a^	0.8 (0.50 to 1.09)	2.22 (1.66 to 2.96)	<.001	0.70 (0.44 to 0.95)	2.00 (1.55 to 2.57)	<.001
Diabetes^a^	0.35 (0.11 to 0.60)	1.42 (1.11 to 1.81)	.005	0.31 (0.09 to 0.52)	1.36 (1.10 to 1.68)	.005
Hypertension^a^	0.35 (0.06 to 0.64)	1.42 (1.07 to 1.89)	.02	0.31 (0.06 to 0.56)	1.36 (1.06 to 1.74)	.01
FIES Categorical household food insecurity						
Food secure	1 [Reference]	1 [Reference]	NA	1 [Reference]	1 [Reference]	NA
Mild to moderate food insecurity	0.5 (–0.08 to 1.08)	1.64 (0.92 to 2.94)	.09	0.43 (–0.07 to 0.94)	1.54 (0.93 to 2.56)	.09
Severe food insecurity	1.21 (0.62 to 1.80)	3.37 (1.87 to 6.07)	<.001	1.06 (0.54 to 1.57)	2.88 (1.72 to 4.81)	<.001
Household water insecurity experiences scale						
Household water insecurity	0.2 (–0.06 to 0.46)	1.22 (0.94 to 1.59)	.14	0.17 (–0.06 to 0.40)	1.19 (0.95 to 1.49)	.14
No household water insecurity	1 [Reference]	1 [Reference]	NA	1 [Reference]	1 [Reference]	NA
Receipt of cash assistance						
No	0.3 (0.05 to 0.55)	1.35 (1.05 to 1.73)	.02	0.26 (0.04 to 0.48)	1.30 (1.04 to 1.61)	.02
Yes	1 [Reference]	1 [Reference]	NA	1 [Reference]	1 [Reference]	NA
Intercept	–2.48 (–3.63 to –1.33)	0.08 (0.03 to 0.26)	<.001	–2.33 (–2.44 to –2.21)	0.10 (0.09 to 0.11)	<.001

Abbreviation: FIES, Food Insecurity Experience Scale.

^a^ Reference category for each self-reported medical condition is no.

All the included predictors had the expected direction coefficient with the outcome. In particular, individuals who did not receive cash assistance, who had household water insecurity, or had severe food insecurity had increased likelihood of being unable to manage their NCDs. Age had a small negative coefficient with inability to manage NCDs.

To illustrate, the predicted risk of inability to manage NCDs for a Syrian refugee aged 60 years with all 4 NCDs, severe household food insecurity, no receipt of cash assistance, and household water insecurity was 51% (using the optimized-adjusted coefficients). While the predicted risk of inability to manage NCDs for a refugee aged 60 years with all 4 NCDs who received cash assistance and was food and water secure was 19% (using the optimized-adjusted coefficients). For a Syrian refugee aged 60 years who had hypertension and diabetes without other NCDs and similar characteristics to the latter individual, the predicted risk was 9%.

In a sensitivity analysis, total number of chronic diseases was modeled as a categorical variable rather than each individual NCD. The predictors identified in this alternative model were the number of chronic diseases, food insecurity, age, and nonreceipt of cash assistance. However, the discrimination (*C* statistic, 0.633; 95% CI, 0.602 to 0.662) and calibration (*C* slope, 0.863; 95% CI, 0.710 to 1.064) of the optimized-adjusted model were poorer than the final model (eTable 5 in the Supplement).

## Discussion

This prognostic study identified predictors of inability to manage NCDs among older Syrian refugees in Lebanon during the COVID-19 pandemic. Younger age, not receiving cash assistance, household water insecurity, severe household food insecurity, and comorbidity were predictors of inability to manage NCDs. The predictive model had a moderate discriminative ability, and the calibration of the model showed overfitting. One of the key barriers for older Syrian refugees to adhere to medication was cost of the medication and beliefs that they did not require the medication anymore after feeling better. In addition, for older Syrian refugees who were unable to access primary health care, the costs of the visit, tests, or medications were the main reasons.

Our study found that Syrian refugees who did not receive cash assistance had a higher likelihood of being unable to manage their NCDs. This finding concurs with another study conducted in Lebanon among Syrian refugees,^34^ which found that receiving multipurpose cash assistance was associated with increased access to primary health care for various illnesses, including chronic diseases. Most Syrian refugees rely on cash assistance to cover their basic needs, such as food, rent, water, and health care.^35^ Hence, one possible explanation is that without cash assistance, resources are limited to the acute and immediate livelihood needs rather than managing a chronic disease. According to the UNHCR, medications for chronic diseases are free, so refugees without cash assistance may not be able to afford transportation to obtain medications. Similarly, studies performed in Lebanon and Jordan among Syrian refugees reported that the costs of treatment and clinic visits were the primary reasons for not seeking health care for chronic diseases.^16,36^ Recently, Lebanon has experienced a shortage in the supply of medications due to the economic crisis.^18^ It remains crucial for humanitarian agencies to remove the barriers to accessing health care and medications for older Syrian refugee populations.

Water insecurity and severe food insecurity among Syrian refugees are markers of severe economic vulnerability and low socioeconomic status.^37,38,39^ As a result, households that are unable to meet their basic needs for food and water are unlikely to be able to use a proportion of their household income for any costs related to accessing medications. Furthermore, food insecurity has been shown to reduce overall dietary quality and diversity.^40,41,42^ Thus, food insecurity can prevent refugees from modifying and improving their diet quality to manage their chronic disease.

Multimorbidity is common in older adults and it has implications on the self-management of chronic conditions.^43^ Our results are consistent with previous research, such as a systematic review by Kardas et al,^44^ that suggested that older adults with multimorbidity have reduced adherence to managing their NCDs. Polypharmacy and complex dosing regimens are common in older age,^45^ and these can reduce adherence,^44^ as each medication may have its own specific instructions to follow,^46^ which can be increasingly difficult in older age.

There was no statistical association between age and self-management of NCDs. Previous studies have shown that younger age was associated with lower medication adherence for depression^47^ and heart failure,^48^ while other studies have suggested no association between age and medication adherence.^49^

There is increasing recognition that management of NCDs among refugee populations represents a challenge for humanitarian agencies, as these are costly to manage with limited resources available for health care.^16^ Further advocacy is required for donor organizations to prioritize NCDs in this population so that the health and well-being of older refugee populations can be maximized. With the recent war in Ukraine and worsening of humanitarian catastrophe, there have been growing calls for immediate and rapid access to medicines for individuals with NCDs.^50^ In this context, reinforcing self-management protocols and health literacy, alleviating food insecurity, and enabling dietary diversity are required to prevent secondary complications of unmanaged NCDs. Moreover, further studies targeting NCD interventions among this vulnerable population in the Middle East are crucial to reducing the disease burden, especially in view of the very high prevalence of these conditions.

### Limitations

This study has some limitations. The study population is representative of the beneficiaries from a large humanitarian organization, but not all older Syrian refugees in Lebanon, so the estimates cannot be used to measure national prevalence (eAppendix in the Supplement). The predictive model had a moderate discriminative ability, which may be explained by missing predictors.^12,36,51,52^ Furthermore, the calibration of the model showed overfitting, and the model may not perform well in future samples; hence, future studies should aim to be larger if they wish to develop a predictive model. The study outcome was subjective and may be prone to misclassification; future studies should aim to measure blood pressure, cholesterol, and fasting glucose or hemoglobin A_1c_ and to collect symptoms and adherence using validated questionnaires. Additionally, efforts must be made to collect complete and accurate data on cardiovascular events and cause of death in humanitarian settings.

## Conclusions

This prognostic study highlights that inability to manage NCDs among Syrian refugees in Lebanon was mainly associated with financial barriers. The predictors identified in this study could allow health care professionals and humanitarian organizations to identify older refugees who are at a greater risk of being unable to manage their NCDs. These vulnerable groups should have the necessary assistance to allow an improvement in medication adherence and equitable access to health care. Furthermore, investment in NCD health care services in primary care will be beneficial to the prevention of premature mortality and morbidity from NCDs in Lebanon and elsewhere.

## Notes

Supplement.
eMethods.
**eAppendix.** Representativeness of the Sample **eTable 1.** Characteristics of Nonparticipants and Participants **eTable 2.** Prevalence of NCDs Amongst the Overall Sample and the Study Population **eTable 3.** The Ability to Manage Condition by NCD Type and Number of NCDs **eTable 4.** Performance of the Apparent and Optimized Adjusted Models **eTable 5. **Sensitivity Analysis Using the Number of Chronic Conditions for the Predictive Model Estimates and Statistics **eFigure.** Calibration Plot of Model Performance in the Apparent Model
eReferences.

## References

[1] World Health Organization (WHO). ApartTogether survey: preliminary overview of refugees and migrants self-reported impact of COVID-19. Accessed February 18, 2021. https://apps.who.int/iris/handle/10665/337931

[2] Bukuluki P, Mwenyango H, Katongole SP, Sidhva D, Palattiyil G. The socioeconomic and psychosocial impact of COVID-19 pandemic on urban refugees in Uganda. Soc Sci Humanit Open. 2020;2(1):100045. doi:10.1016/j.ssaho.2020.100045 34173490 PMC7351411

[3] Fouad FM, McCall SJ, Ayoub H, Abu-Raddad LJ, Mumtaz GR. Vulnerability of Syrian refugees in Lebanon to COVID-19: quantitative insights. Confl Health. 2021;15(1):13. doi:10.1186/s13031-021-00349-6 33673855 PMC7934989

[4] Rehr M, Shoaib M, Ellithy S, . Prevalence of non-communicable diseases and access to care among non-camp Syrian refugees in northern Jordan. Confl Health. 2018;12(1):33. doi:10.1186/s13031-018-0168-7 30008800 PMC6040066

[5] Wang B, Li R, Lu Z, Huang Y. Does comorbidity increase the risk of patients with COVID-19: evidence from meta-analysis. Aging (Albany NY). 2020;12(7):6049-6057. doi:10.18632/aging.103000 32267833 PMC7185114

[6] Akik C, Ghattas H, Mesmar S, Rabkin M, El-Sadr WM, Fouad FM. Host country responses to non-communicable diseases amongst Syrian refugees: a review. Confl Health. 2019;13:8. doi:10.1186/s13031-019-0192-2 30949232 PMC6431037

[7] Demaio A, Jamieson J, Horn R, de Courten M, Tellier S. Non-communicable diseases in emergencies: a call to action. PLoS Curr. 2013;5:5. doi:10.1371/currents.dis.53e08b951d59ff913ab8b9bb51c4d0de 24056956 PMC3775888

[8] WHO. Adherence to long term therapies: Evidence for action. Published 2003. Accessed February 18, 2021. http://apps.who.int/iris/bitstream/handle/10665/42682/9241545992.pdf;jsessionid=6B7994A65CA1BBDE3B6CB3ACDEDA4F49?sequence=1

[9] Unal B, Critchley JA, Capewell S. Explaining the decline in coronary heart disease mortality in England and Wales between 1981 and 2000. Circulation. 2004;109(9):1101-1107. doi:10.1161/01.CIR.0000118498.35499.B2 14993137

[10] Capewell S, Beaglehole R, Seddon M, McMurray J. Explanation for the decline in coronary heart disease mortality rates in Auckland, New Zealand, between 1982 and 1993. Circulation. 2000;102(13):1511-1516. doi:10.1161/01.CIR.102.13.1511 11004141

[11] Nelson MR, Reid CM, Ryan P, Willson K, Yelland L. Self-reported adherence with medication and cardiovascular disease outcomes in the Second Australian National Blood Pressure Study (ANBP2). Med J Aust. 2006;185(9):487-489. doi:10.5694/j.1326-5377.2006.tb00662.x 17137452

[12] Mohamad M, Moussally K, Lakis C, . Self-reported medication adherence among patients with diabetes or hypertension, Médecins Sans Frontières Shatila refugee camp, Beirut, Lebanon: a mixed-methods study. PLoS One. 2021;16(5):e0251316. doi:10.1371/journal.pone.0251316 33970972 PMC8109801

[13] Strong J, Varady C, Chahda N, Doocy S, Burnham G. Health status and health needs of older refugees from Syria in Lebanon. Confl Health. 2015;9:12. doi:10.1186/s13031-014-0029-y 26056531 PMC4459463

[14] UNHCR. Health programme. Accessed February 16, 2021. https://www.unhcr.org/lb/wp-content/uploads/sites/16/2019/04/Health-Factsheet.pdf

[15] DeJong J, Ghattas H, Bashour H, Mourtada R, Akik C, Reese-Masterson A. Reproductive, maternal, neonatal and child health in conflict: a case study on Syria using Countdown indicators. BMJ Glob Health. 2017;2(3):e000302. doi:10.1136/bmjgh-2017-000302 29225945 PMC5717942

[16] Doocy S, Lyles E, Akhu-Zaheya L, Burton A, Burnham G. Health service access and utilization among Syrian refugees in Jordan. Int J Equity Health. 2016;15(1):108. doi:10.1186/s12939-016-0399-4 27418336 PMC4946096

[17] World Health Organization. COVID-19 significantly impacts health services for noncommunicable diseases. Accessed February 28, 2022. https://www.who.int/news-room/detail/01-06-2020-covid-19-significantly-impacts-health-services-for-noncommunicable-diseases

[18] Ahsan S. Lebanese health care racked by medicine shortages. Lancet. 2021;398(10300):568. doi:10.1016/S0140-6736(21)01852-3 34391489

[19] Altare C, Kostandova N, OKeeffe J, . COVID-19 epidemiology and changes in health service utilization in Azraq and Zaatari refugee camps in Jordan: a retrospective cohort study. PLoS Med. 2022;19(5):e1003993. doi:10.1371/journal.pmed.1003993 35536871 PMC9089859

[20] Women U. Rapid gender assessments on the socioeconomic impacts of COVID-19. Accessed June 28, 2022. https://data.unwomen.org/rga

[21] Abdulrahim A, Ghattas H, McCall S. Changing vulnerabilities and COVID-19 adherence: older refugees in Lebanon. Accessed February 23, 2022. https://www.elrha.org/project/covid-19-adherence-older-refugees-lebanon/

[22] Jeste DV, Palmer BW, Appelbaum PS, . A new brief instrument for assessing decisional capacity for clinical research. Arch Gen Psychiatry. 2007;64(8):966-974. doi:10.1001/archpsyc.64.8.966 17679641

[23] Abdulrahim S, Ghattas H, McCall S, . Tracking adherence of older refugees to COVID-19 preventive measures in response to changing vulnerabilities: a multi-level, panel study to inform humanitarian response in Lebanon, 2021 [survey documentation]. Accessed August 15, 2022. https://scholarworks.aub.edu.lb/handle/10938/22852

[24] Cafiero C, Viviani S, Nord M. Food security measurement in a global context: the food insecurity experience scale. Measurement. 2018;116:146-152. doi:10.1016/j.measurement.2017.10.065

[25] Young SL, Miller JD, Frongillo EA, Boateng GO, Jamaluddine Z, Neilands TB; HWISE Research Coordination Network. Validity of a four-item household water insecurity experiences scale for assessing water issues related to health and well-being. Am J Trop Med Hyg. 2021;104(1):391-394. doi:10.4269/ajtmh.20-0417 33124535 PMC7790094

[26] Harrell FE. Multivariable modeling strategies. In: Regression Modeling Strategies. Springer; 2015:63-102. doi:10.1007/978-3-319-19425-7_4

[27] Sauerbrei W. The use of resampling methods to simplify regression models in medical statistics. J R Stat Soc Ser C Appl Stat. 1999;48(3):313-329. doi:10.1111/1467-9876.00155

[28] Van Calster B, McLernon DJ, van Smeden M, Wynants L, Steyerberg EW; Topic Group ‘Evaluating diagnostic tests and prediction models’ of the STRATOS initiative. Calibration: the Achilles heel of predictive analytics. BMC Med. 2019;17(1):230. doi:10.1186/s12916-019-1466-7 31842878 PMC6912996

[29] Steyerberg EW. Overfitting and optimism in prediction models. In: Clinical Prediction Models. Springer; 2019:95-112. doi:10.1007/978-3-030-16399-0_5

[30] Leijdekkers JA, Eijkemans MJC, van Tilborg TC, ; OPTIMIST group. Predicting the cumulative chance of live birth over multiple complete cycles of in vitro fertilization: an external validation study. Hum Reprod. 2018;33(9):1684-1695. doi:10.1093/humrep/dey263 30085143

[31] Harrell FE Jr, Lee KL, Mark DB. Multivariable prognostic models: issues in developing models, evaluating assumptions and adequacy, and measuring and reducing errors. Stat Med. 1996;15(4):361-387. doi:10.1002/(SICI)1097-0258(19960229)15:4<361::AID-SIM168>3.0.CO;2-4 8668867

[32] Fernandez-Felix B, García-Esquinas E, Muriel A, Royuela A, Zamora J. Bootstrap internal validation command for predictive logistic regression models. Stata J. 2021;21(2):498-509. doi:10.1177/1536867X211025836

[33] Moons KG, Donders ART, Steyerberg EW, Harrell FE. Penalized maximum likelihood estimation to directly adjust diagnostic and prognostic prediction models for overoptimism: a clinical example. J Clin Epidemiol. 2004;57(12):1262-1270. doi:10.1016/j.jclinepi.2004.01.020 15617952

[34] Chaaban J, Salti N, Ghattas H, . Multi-purpose cash assistance in Lebanon: impact evaluation on the well-being of Syrian refugees. Accessed August 15, 2022. https://www.nrc.no/globalassets/pdf/reports/camealeon-impact-assessment-of-multi-purpose-cash-assistance-for-syrian-refugees-in-lebanon/camealeon-mpc-impact-assessment.pdf

[35] Ratnayake R, Rawashdeh F, AbuAlRub R, . Access to care and prevalence of hypertension and diabetes among Syrian refugees in northern Jordan. JAMA Netw Open. 2020;3(10):e2021678-e2021678. doi:10.1001/jamanetworkopen.2020.21678 33052405 PMC7557515

[36] Lyles E, Burnham G, Chlela L, Spiegel P, Morlock L, Doocy S; Lebanon Health Access Survey (LHAS) Study Team. Health service utilization and adherence to medication for hypertension and diabetes among Syrian refugees and affected host communities in Lebanon. J Diabetes Metab Disord. 2020;19(2):1245-1259. doi:10.1007/s40200-020-00638-6 32963978 PMC7498301

[37] Workman CL, Brewis A, Wutich A, Young S, Stoler J, Kearns J. Understanding biopsychosocial health outcomes of syndemic water and food insecurity: applications for global health. Am J Trop Med Hyg. 2021;104(1):8-11. doi:10.4269/ajtmh.20-0513 33146108 PMC7790089

[38] Jamaluddine Z, Sahyoun NR, Choufani J, Sassine AJ, Ghattas H. Child-reported food insecurity is negatively associated with household food security, socioeconomic status, diet diversity, and school performance among children attending UN Relief and Works Agency for Palestine Refugees schools in Lebanon. J Nutr. 2019;149(12):2228-2235. doi:10.1093/jn/nxz189 31504697

[39] Omidvar N, Ghazi-Tabatabie M, Sadeghi R, Mohammadi F, Abbasi-Shavazi MJ. Food insecurity and its sociodemographic correlates among Afghan immigrants in Iran. J Health Popul Nutr. 2013;31(3):356-366. doi:10.3329/jhpn.v31i3.16828 24288950 PMC3805886

[40] Leung CW, Epel ES, Ritchie LD, Crawford PB, Laraia BA. Food insecurity is inversely associated with diet quality of lower-income adults. J Acad Nutr Diet. 2014;114(12):1943-53.e2. doi:10.1016/j.jand.2014.06.353 25091796

[41] Ghattas H, Sassine AJ, Seyfert K, Nord M, Sahyoun NR. Food insecurity among Iraqi refugees living in Lebanon, 10 years after the invasion of Iraq: data from a household survey. Br J Nutr. 2014;112(1):70-79. doi:10.1017/S0007114514000282 24739803

[42] Seligman HK, Laraia BA, Kushel MB. Food insecurity is associated with chronic disease among low-income NHANES participants. J Nutr. 2010;140(2):304-310. doi:10.3945/jn.109.112573 20032485 PMC2806885

[43] Barnett K, Mercer SW, Norbury M, Watt G, Wyke S, Guthrie B. Epidemiology of multimorbidity and implications for health care, research, and medical education: a cross-sectional study. Lancet. 2012;380(9836):37-43. doi:10.1016/S0140-6736(12)60240-2 22579043

[44] Kardas P, Lewek P, Matyjaszczyk M. Determinants of patient adherence: a review of systematic reviews. Front Pharmacol. 2013;4:91. doi:10.3389/fphar.2013.00091 23898295 PMC3722478

[45] Linjakumpu T, Hartikainen S, Klaukka T, Veijola J, Kivelä S-L, Isoaho R. Use of medications and polypharmacy are increasing among the elderly. J Clin Epidemiol. 2002;55(8):809-817. doi:10.1016/S0895-4356(02)00411-0 12384196

[46] Ingersoll KS, Cohen J. The impact of medication regimen factors on adherence to chronic treatment: a review of literature. J Behav Med. 2008;31(3):213-224. doi:10.1007/s10865-007-9147-y 18202907 PMC2868342

[47] Bambauer KZ, Soumerai SB, Adams AS, Zhang F, Ross-Degnan D. Provider and patient characteristics associated with antidepressant nonadherence: the impact of provider specialty. J Clin Psychiatry. 2007;68(6):867-873. doi:10.4088/JCP.v68n0607 17592910

[48] Bagchi AD, Esposito D, Kim M, Verdier J, Bencio D. Utilization of, and adherence to, drug therapy among Medicaid beneficiaries with congestive heart failure. Clin Ther. 2007;29(8):1771-1783. doi:10.1016/j.clinthera.2007.08.015 17919558

[49] Krueger K, Botermann L, Schorr SG, Griese-Mammen N, Laufs U, Schulz M. Age-related medication adherence in patients with chronic heart failure: a systematic literature review. Int J Cardiol. 2015;184:728-735. doi:10.1016/j.ijcard.2015.03.042 25795085

[50] Ioffe Y, Abubakar I, Issa R, Spiegel P, Kumar BN. Meeting the health challenges of displaced populations from Ukraine. Lancet. 2022;399(10331):1206-1208. doi:10.1016/S0140-6736(22)00477-9 35286844 PMC8916778

[51] Chew SM, Lee JH, Lim SF, Liew MJ, Xu Y, Towle RM. Prevalence and predictors of medication nonadherence among older community-dwelling people with chronic disease in Singapore. J Adv Nurs. 2021;77(10):4069-4080. doi:10.1111/jan.14913 34061364

[52] Nair KV, Belletti DA, Doyle JJ, . Understanding barriers to medication adherence in the hypertensive population by evaluating responses to a telephone survey. Patient Prefer Adherence. 2011;5:195-206. doi:10.2147/PPA.S18481 21573051 PMC3090381

