# Supplementary material for: Development of a Prediction Model for the Management of Noncommunicable Diseases Among Older Syrian Refugees Amidst the COVID-19 Pandemic in Lebanon
Source: JAMA Netw Open. 2022 Oct 13;5(10):e2231633. doi: 10.1001/jamanetworkopen.2022.31633 (PMC9561955; doi:10.1001/jamanetworkopen.2022.31633)
Supplement: Supplement. — eMethods. eAppendix. Representativeness of the Sample eTable 1. Characteristics of Nonparticipants and Participants eTable 2. Prevalence of NCDs Amongst the Overall Sample and the Study Population eTable 3. The Ability to Manage Condition by NCD Type and Number of NCDs eTable 4. Performance of the Apparent and Optimized Adjusted Models eTable 5. Sensitivity Analysis Using the Number of Chronic Conditions for the Predictive Model Estimates and Statistics eFigure. Calibration Plot of Model Performance in the Apparent Model eReferences. [file jamanetwopen-e2231633-s001.pdf]

Supplemental Online Content

McCall SJ, El Khoury T, Salibi N, et al. Development of a prediction model for the management of noncommunicable diseases among older Syrian refugees amidst the COVID-19 pandemic in Lebanon. *JAMA Netw Open*. 2022;5(10):e2231633. doi:10.1001/jamanetworkopen.2022.31633

eMethods.

eAppendix. Representativeness of the Sample

eTable 1. Characteristics of Nonparticipants and Participants

eTable 2. Prevalence of NCDs Amongst the Overall Sample and the Study Population

eTable 3. The Ability to Manage Condition by NCD Type and Number of NCDs

eTable 4. Performance of the Apparent and Optimized Adjusted Models

eTable 5. Sensitivity Analysis Using the Number of Chronic Conditions for the Predictive Model Estimates and Statistics

eFigure. Calibration Plot of Model Performance in the Apparent Model

eReferences.

This supplemental material has been provided by the authors to give readers additional information about their work.

© 2022 McCall SJ et al. *JAMA Network Open.*

eMethods.

Study design and sampling

This was a cross-sectional study nested within a multi-wave rotating longitudinal study, entitled "Tracking Adherence of Older Refugees to COVID-19 Preventive Measures in Response to Changing Vulnerabilities: A Multi Level, Panel Study to Inform Humanitarian Response in Lebanon".

All beneficiaries' households (n=17, 384) who received assistance/services from the humanitarian organization between 2017 and 2020 and included at least one adult aged 50 years or older were contacted by telephone. Multiple call attempts at different times of the day were made to maximise the response rate. For each household, a roster of eligible participants was created, then only one eligible adult, aged 50 years or older was randomly selected, using a computer algorithm in KoBotoolbox, and invited to consent. Among consented households, 71.4% had only one older adult, 27.3% had two older adults and 1.3% had more than 3 older adults.

Prior to consent, individuals aged 65 years or older were assessed for their capacity to consent, using five modified items from the University of California, San Diego Brief Assessment of Capacity to Consent[1]. Notably, these five questions were chosen and modified as relevant to the study. The questions used were: (1) What is the purpose of the study that was just described to you?; (2) Do you think you are participating in a study or in an evaluation of services/humanitarian aid?; (3) If you withdraw from this study, will you still be able to receive regular benefits?; (4) If you participate in this study, what are some of the things that you will be asked to do? ; (5) Is it possible that being in this study will not have any direct benefit to you? Each item was rated on a Likert scale from 0 (little to no understanding) to 2 (clear understanding). Therefore, participants who had a score of 7 out 10 were considered as able to participate.

Development of the survey tool

The survey tool was drafted in English, then back translated into Arabic. The instrument included many modules, such as sociodemographic characteristics, health, COVID-19, shelter, household water insecurity, safety and security, social support, violence and trauma, decision making, communication, assets, expenditure, assistance, income, debt, food security, and regularization. Notably, modules varied between waves. The full questionnaires and modules are available online [link:https://scholarworks.aub.edu.lb/handle/10938/22852][2].

Following extensive literature review, the survey tool was first drafted by a group of academics who have experience in survey methods and have expertise in humanitarian settings, and migrant and refugee health. It included many validated tools such as Coping Strategies Index, Food Insecurity Experience Scale (FIES)[3], Household Water Insecurity Experiences Scale (HWISE-4)[4], 20-Item Short Form Survey Instrument (SF-20)[5], Mental Health Inventory Scale (MHI-5)[6] , and the Washington Group Disability Scale[7]. The tool was then internally reviewed by academics and humanitarian actors (different departments and sector leads) through multiple consultations to ensure the reliability and usefulness of the tool. The program specialists (water, protection, legal, shelter etc.) within NRC provided questions that would be useful for informing humanitarian programming and they reviewed the survey tool. Once consensus was reached on the different sections of the tool, community consultations with beneficiaries, community focal points and other stakeholders (e.g., Municipality members, Mukhtars, community leaders) took place in three regions in Lebanon (North, Bekaa and South). In these consultations we received in-depth community feedback on the overall study and specific modules/questions within the survey tool. The community consultations allowed the team to better understand the acceptability and validity of the modules included, and whether they met the needs of the beneficiaries. Beneficiaries and other community stakeholders had valuable contributions regarding what factors, needs and vulnerabilities were important to explore. Based on the feedback received, the survey tool was adjusted to capture contextually-relevant perceptions and experiences that were not previously included.

Prior to data collection, a three-day training was delivered to data collectors on the survey tool, humanitarian and research ethics, communication techniques, use of tablets, as well as the referral mechanisms for participants requiring any humanitarian assistance. A one-day refresher training was also given prior to each wave. The survey tool was piloted to assess face validity amongst the data collectors and to capture any errors in the survey tool.

The Arabic version was pilot tested among a random sample of 6 Syrian refugees aged 50 years and older. Several updates were made to the survey instrument following community feedback, data collector training, and the pilot test to ensure that the Arabic version was culturally and linguistically appropriate.

We used computer-assisted telephone interviewing (CATI) where the survey tool was programmed on KoBoToolbox and the interviewer entered the respondent's answers into an electronic form. This data collection platform included logical skip

© 2022 McCall SJ et al. *JAMA Network Open.*

patterns and validation minimizing data entry errors. It also had quick basic data analysis and visualization features, which allowed for real-time monitoring of collected data. During data collection, recordings of the initial interviews for each data collector were listened to and feedback was given to the data collectors. In addition, two research assistants were regularly exporting the data, and ran data monitoring checks to ensure data quality. A weekly meeting was also held with the data collection company to follow up on the data collection process and raise any concerns. The data collection company completed call back checks with a subset of participants each week and randomly checked values entered into the questionnaire. If there were impossible values or missing data on key variables these participants were recalled by the data collection company to check these values and ascertain if the missing items could be completed.

© 2022 McCall SJ et al. *JAMA Network Open*.

eAppendix. Representativeness of the Sample

Representativeness of sample

This study used the entire listing of households with an identified older adult from a large NGO in Lebanon (Norwegian Refugee Council (NRC)) as a sampling frame, as a result, this study will be representative of the NRC's beneficiaries. NRC provides humanitarian assistance in the form of shelter, protection, legal assistance, water, hygiene and sanitation (WASH) and education. This study cannot be used to estimate the national prevalence of NCDs for older Syrian refugees in Lebanon. Each year the UNHCR, UNICEF and WFP conduct a nationally representative survey of Syrian refugees households in Lebanon and this sample includes Syrian refugees of all ages so it cannot be directly compared with this study's sample.[8] In addition, a sample of older Syrian refugees are likely to be more vulnerable than a sample of Syrian refugees of all ages. Similar to VASrY, the largest proportions of refugees in the study population where from the Bekaa and the North of Lebanon. In addition, there were comparable proportion of households with family debts (95% in study population vs. 92% in VASyR, 2021). Receipt of cash or voucher assistance was broadly similar; however, they were collected at different time points and cash assistance increased from 47% in August 2020 to 73% in June 2021 (71% in study population (September 2020-January 2021); 73% in VASrY June-July, 2021 (ATM cards and E-cards)).[8]

© 2022 McCall SJ et al. *JAMA Network Open*.

eTable 1. Characteristics of Nonparticipants and Participants

Assessment for capacity to consent (age ≥65 years)	Excluded (n=129)		Included (n=746)			
Age, Median (IQR)	69	(67-72)	70	(67-74)		
**Consent pre-wave 1**	**Refused (n=43)**		**Accepted (n=3,838)**			
Age, Median (IQR)	60	(54-65)	56	(53-63)		
**Participation in wave 2**	**Loss to follow up[a] (n=516)**		**Participated in wave 2 (n=3,322)**		**Study population (n=1,893)**	
Age, Median (IQR)	57	(53-63)	56	(53-62)	59	(54-65)
Sex, No. (%)						
Female	256	(50.4)	1584	(52.3)	1,089	(57.5)
Male	260	(49.6)	1738	(47.7)	804	(42.5)
Governorate, No. (%)						
Beirut/Mount Lebanon	10	(1.9)	49	(1.5)	28	(1.5)
Beqaa/Baalbek-Hermel	227	(44.0)	1527	(46.0)	826	(43.6)
North/Akkar	170	(32.9)	1185	(35.7)	727	(38.4)
South/Nabatieh	109	(21.1)	561	(16.9)	312	(16.5)
Residence, No. (%)						
Inside informal tented settlements	188	(36.4)	1286	(38.7)	689	(36.4)
Outside informal tented settlements	328	(63.6)	2036	(61.3)	1,204	(63.6)
Living arrangement, No. (%)						
Living alone	6	(1.2)	62	(1.9)	34	(1.8)
Living with others	510	(98.8)	3260	(98.1)	1,859	(98.2)
Level of Education, No. (%)						
Elementary	113	(22.0)	857	(25.9)	443	(23.4)
Never attended school	273	(53.2)	1611	(48.6)	1,013	(53.6)
Preparatory & Secondary+	127	(24.8)	846	(25.5)	434	(23.0)
Missing	3		8		3	
Family debts, No. (%)						
No	29	(5.7)	237	(7.2)	71	(3.8)
Yes	481	(94.3)	3067	(92.8)	1,817	(96.2)
Missing	6		18		5	
Receipt of cash or voucher assistance, No. (%)						
No	176	(34.2)	1003	(30.2)	576	(30.5)
Yes	338	(65.8)	2314	(69.8)	1,315	(69.5)
Missing	2		5		2	
FIES Categorical household food insecurity, No. (%)						
Food secure	58	(11.8)	259	(8.1)	148	(8.0)
Mild to moderate food insecurity	198	(40.2)	1506	(47.0)	1,071	(57.6)
Severe food insecurity	237	(48.1)	1436	(44.9)	640	(34.4)
Missing	23		121		34	
Household Water Insecurity Experiences Scale, No. (%)						
Household water insecurity	167	(32.5)	1020	(30.8)	515	(27.3)
No household water insecurity	347	(67.5)	2291	(69.2)	1,372	(72.7)
Missing	2		11		6	

[a]Participated in wave 1 and did not participate in wave 2.

© 2022 McCall SJ et al. *JAMA Network Open.*

eTable 2. Prevalence of NCDs Amongst the Overall Sample and the Study Population

	Overall (n=3322)		Study population (n=1893)	
**Type of Non-communicable disease**	**n**	**n (%)**	**n**	**n (%)**
**Hypertension**	3297	1394 (42.3)	1886	1388 (73.6)
Missing	25		7	
**Diabetes**	3306	784 (23.7)	1886	781 (41.4)
Missing	16		7	
**History of cardiovascular disease**	3299	795 (24.1)	1883	794 (42.2)
Missing	23		10	
**Chronic respiratory disease**	3308	351 (10.6)	1888	351 (18.6)
Missing	14		5	

© 2022 McCall SJ et al. *JAMA Network Open*.

eTable 3. The Ability to Manage Condition by NCD Type and Number of NCDs

Type of NCD[a]	Ability to manage NCD	
	Yes, No. (%)	No, No. (%)
Hypertension	972 (84)	185 (16)
Diabetes	547 (83.5)	108 (16.5)
CVD	568 (82.1)	124 (17.9)
Chronic respiratory disease	189 (73.5)	68 (26.5)

Number of NCD	Able to manage NCD(s)				
	None	One NCD	Two NCD	Three NCD	Four NCD
Has one NCD	67 (13.9)	415 (86.1)	X	X	X
Has two NCD	113 (15.4)	307 (41.9)	313 (42.7)	X	X
Has three NCD	49 (11.5)	66 (15.4)	191 (44.6)	122 (28.5)	X
Has four NCDs	36 (13.7)	37 (14.1)	54 (20.6)	110 (42.0)	25 (9.6)

[a] NCD: Non communicable disease

© 2022 McCall SJ et al. *JAMA Network Open.*

eTable 4. Performance of the Apparent and Optimized Adjusted Models

Parameter	Estimate (95%CI)	
	Apparent model	Model adjusted by bootstrap shrinkage
C statistic	0.670 (0.638 to 0.701)	0.650 (0.620 to 0.676)
C slope	1.000 (0.805 to 1.195)	0.871 (0.729 to 1.023)
Calibration-in-the-large	0.000 (–0.119 to 1.119)	0.003 (–0.112 to 0.126)

© 2022 McCall SJ et al. *JAMA Network Open*.

eTable 5. Sensitivity Analysis Using the Number of Chronic Conditions for the Predictive Model Estimates and Statistics

	Apparent model			Bootstrap shrinkage adjusted model		
	**Parameter estimate (95% CI)**	**Odds Ratio (95%CI)**	**P-value**	**Parameter estimate (95% CI)**	**Odds Ratio (95% CI)**	**P-value**
Age	-0.01 (-0.03 to 0.00)	0.99 (0.97 to 1.00)	.09	-0.01 (-0.03 to 0.001)	0.99 (0.97 to 1.00)	.09
Number of chronic conditions						
1		1				
2	0.3 (-0.04 to 0.63)	1.34 (0.96 to1.88)	.08	0.26 (-0.03 to 0.54)	1.29 (0.97 to 1.72)	.08
3 or more	0.77 (0.44 to 1.10)	2.16 (1.55 to 3.00)	<.001	0.66 (0.38 to 0.95)	1.94 (1.46 to 2.58)	<.001
FIES Categorical household food insecurity						
Food secure		1				
Mild to moderate	0.52 (-0.06 to 1.10)	1.68 (0.94 to 3.01)	.08	0.45 (-0.05 to 0.95)	1.57 (0.95 to 2.59)	.07
Severe food insecurity	1.19 (0.61 to 1.78)	3.3 (1.84 to 5.92)	<.001	1.03 (0.53 to 1.53)	2.80 (1.69 to 4.64)	<.001
Receipt of cash or voucher assistance						
No	0.28 (0.03 to 0.53)	1.32 (1.03 to 1.70)	.03	0.24 (0.03 to 0.46)	1.27 (1.03 to 1.58)	.02
Yes		1				
Intercept	-1.85 (-2.96 to -0.74)	0.16 (0.05 to 0.48)	<.001	-1.79 (-1.90 to 1.67)	0.17 (0.15 to 0.19)	<0.001
**Calibration and discrimination of models**						
	**Estimate (95% CI)**			**Estimate (95% CI)**		
C-statistic /Area under the curve	0.652 (0.621 to 0.683)			0.633 (0.602 to 0.662)		
C-slope	0 (-0.118 to 0.118)			0.863 (0.710 to 1.064)		
Calibration in the large	1 (0.778 to 1.222)			0.006 (-0.106 to 1.26)		

© 2022 McCall SJ et al. *JAMA Network Open.*

eFigure. Calibration Plot of Model Performance in the Apparent Model

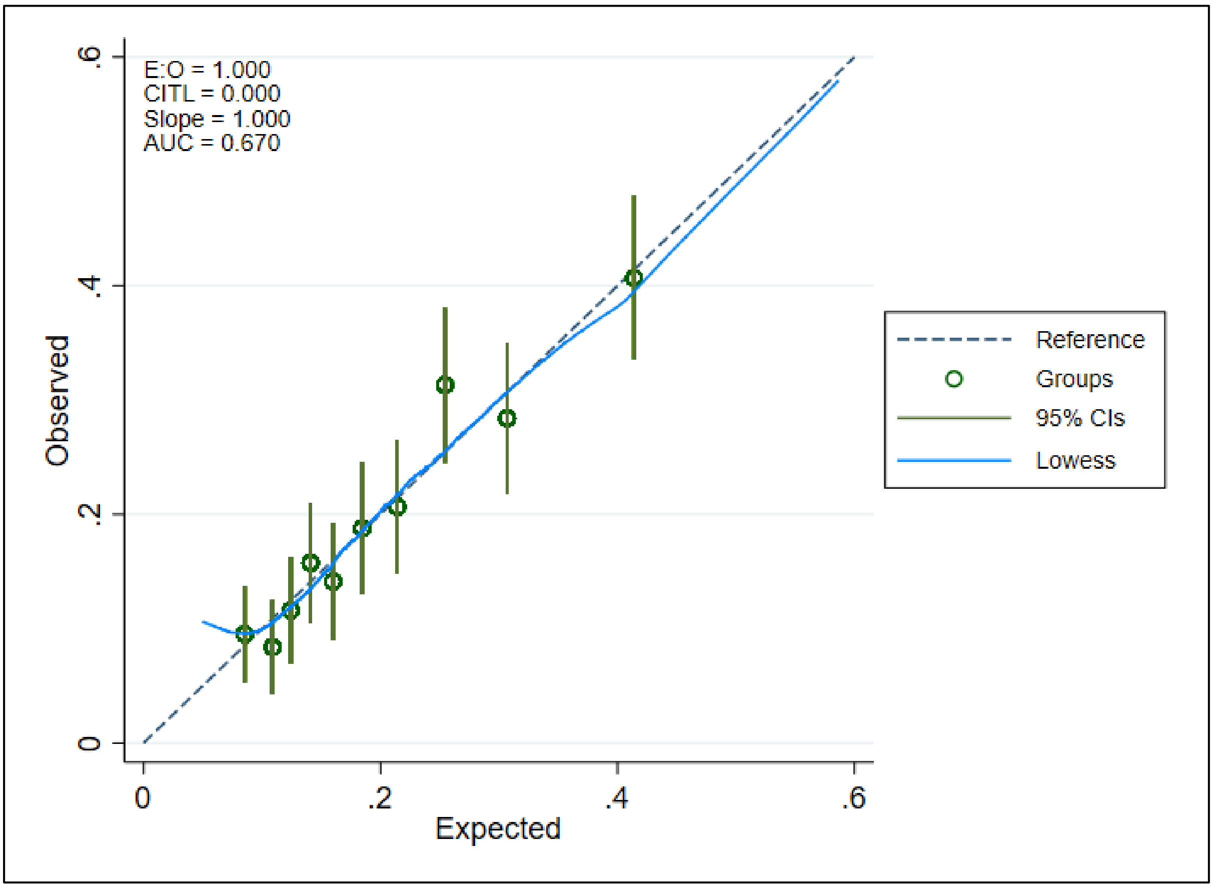

© 2022 McCall SJ et al. *JAMA Network Open*.

eReferences.

1. Jeste DV, Palmer BW, Appelbaum PS, et al. A new brief instrument for assessing decisional capacity for clinical research. *Arch Gen Psychiatry.* 2007;64(8):966-974.
2. Abdulrahim S, Ghattas H, McCall S, et al. Tracking adherence of older refugees to COVID-19 preventive measures in response to changing vulnerabilities: A multi-level, panel study to inform humanitarian response in Lebanon, 2021 [Survey Documentation]. 2021.
3. Cafiero C, Viviani S, Nord M. Food security measurement in a global context: The food insecurity experience scale. *Measurement.* 2018;116:146-152.
4. Young SL, Miller JD, Frongillo EA, Boateng GO, Jamaluddine Z, Neilands TB. Validity of a four-item household water insecurity experiences scale for assessing water issues related to health and well-being. *The American journal of tropical medicine and hygiene.* 2021;104(1):391.
5. Carver DJ, Chapman CA, Thomas VS, Stadnyk KJ, Rockwood K. Validity and reliability of the Medical Outcomes Study Short Form-20 questionnaire as a measure of quality of life in elderly people living at home. *Age Ageing.* 1999;28(2):169-174.
6. Berwick DM, Murphy JM, Goldman PA, Ware JE, Jr., Barsky AJ, Weinstein MC. Performance of a five-item mental health screening test. *Med Care.* 1991;29(2):169-176.
7. Madans JH, Loeb ME, Altman BM. Measuring disability and monitoring the UN Convention on the Rights of Persons with Disabilities: the work of the Washington Group on Disability Statistics. *BMC Public Health.* 2011;11 Suppl 4(Suppl 4):S4.
8. VASyR 2021. Vulnerability Assessment of Syrian Refugees in Lebanon. UNHCR, UNICEF and WFP. https://data2.unhcr.org/en/documents/details/90589. Published 2021. Accessed 30-06-2022, 2022.

© 2022 McCall SJ et al. *JAMA Network Open.*
